# Integrating GPCR-specific information with full text articles

**DOI:** 10.1186/1471-2105-12-362

**Published:** 2011-09-12

**Authors:** Bas Vroling, David Thorne, Philip McDermott, Teresa K Attwood, Gert Vriend, Steve Pettifer

**Affiliations:** 1 CMBI, NCMLS, Radboud University Nijmegen Medical Centre, Geert Grooteplein 26-28, Nijmegen, 6525 GA, The Netherlands; 2 School of Computer Science, University of Manchester, Kilburn Building, Oxford Road, Manchester, M13 9PL, UK; 3 Faculty of Life Sciences, University of Manchester, Michael Smith Building, Oxford Road, Manchester, M13 9PT, UK

## Abstract

**Background:**

With the continued growth in the volume both of experimental G protein-coupled receptor (GPCR) data and of the related peer-reviewed literature, the ability of GPCR researchers to keep up-to-date is becoming increasingly curtailed.

**Results:**

We present work that integrates the biological data and annotations in the GPCR information system (GPCRDB) with next-generation methods for intelligently exploring, visualising and interacting with the scientific articles used to disseminate them. This solution automatically retrieves relevant information from GPCRDB and displays it both within and as an adjunct to an article.

**Conclusions:**

This approach allows researchers to extract more knowledge more swiftly from literature. Importantly, it allows reinterpretation of data in articles published before GPCR structure data became widely available, thereby rescuing these valuable data from long-dormant sources.

## Background

Recent decades have seen a massive increase in the amount of data available to researchers. Revolutionary high-throughput technologies have enabled data production on an unprecedented scale in many areas of the life sciences. To accommodate this increase in experimental data, we have also witnessed a database explosion, with both the number and size of specialised biological repositories growing enormously over the years [1]. Most of these resources offer their own data formats, websites and programmatic interfaces. This has made the task of retrieving information increasingly difficult and complex [2].

GPCRDB [3] is one of the earliest examples of a Molecular-class Specific Information System (MCSIS). It collects, combines, validates and disseminates large amounts of GPCR-related content, bringing together information (such as sequences, structures, mutations and oligomers) from other databases, and augmenting this with manually annotated data (such as mutations extracted from literature), as well as computationally-derived multiple sequence alignments and homology models. Its contents are validated, integrated, internally consistent [4] and updated regularly [3]. GPCRDB thus functions as a one-stop shop for GPCR-related knowledge, relieving scientists of the burden of going through the process of finding multiple data sources, of learning how to use them, and of then retrieving, synthesising and integrating the retrieved information.

Much of the routine practice of communicating scientific knowledge is conducted through the process of reading and writing scientific papers, an invaluable method that has been used successfully for hundreds of years. However, it is now widely acknowledged that this approach imposes considerable constraints upon the type and quantity of biological information published: specifically, the data used to generate hypotheses and to perform experiments are not readily accessible to fellow researchers who use the results of their peers' publications.

Relating information between databases and literature has become increasingly difficult with the growing quantities of data and documents available, and it is evident that new solutions are required to address this problem [5,6]. When reading about a mutation, a scientist might, for example, want to know the location of the mutated residue in the structure, or find other articles describing the same mutation. Although systems like GPCRDB provide scientists with access to focused information derived from the literature, subsequent exploration of concepts in related articles still poses practical hurdles (e.g., opening a browser window, navigating to the database, searching for the data).

With the goal of bridging the gap between data and scientific articles, several initiatives have explored how to enrich scientific literature with repository data, either by manual annotation [7-9] or by automatic approaches [10,11]. The results of these projects are encouraging, but also point to what remains to be done. For example, Shotton *et al. *[9] have explored the use of semantic annotations, beautifully illustrating the possibilities of these types of technologies. However, providing enhancements of this quality on a routine basis is still far beyond our reach; this impressive example is limited to one paper, and a very large amount of manual input was involved. One of the most promising automated initiatives, the Reflect [11] system, provides annotations for a very large number of biological entities, covering genes, proteins and a large number of small-molecule compounds. These annotations contain an enormous wealth of data and hyperlinks to a multitude of information systems. However, automation of the process carries the price of a large number of errors; hence, vigilance and caution are required, coupled with a significant amount of manual effort to validate and disambiguate the results. In addition, the aforementioned approaches all focus on HTML/XML formats, and require users to read their articles in a Web browser, ignoring the *de facto *standard format of scientific literature: Adobe's Portable Document Format (PDF). This is by far the most popular format for peer-reviewed science communication, for reasons that include the benefits of their permanence, device independence, and ubiquitous support and good readability. It therefore makes sense to exploit the PDF; the software tool Utopia Documents [12,13] was developed for just this purpose. Behaving like a familiar PDF reader, Utopia Documents is a desktop application for reading and exploring papers. Its strength lies in being able to semantically annotate concepts in documents with additional relevant information and links to online resources.

We report here a technology that allows on-the-fly annotation of GPCR-related PDF articles. By fully integrating the Utopia Documents PDF reader and the GPCRDB information system, we can present to the scientist, in a non-intrusive way, all possible data and information related to the topics discussed in the article at hand. This makes possible a paradigmatic change in the relationship between the PDF-reading scientist and Internet-based data resources, alleviating the troubles associated with navigating the many links between existing data and information in this field. The scientist neither struggles to get access to information related to topics within an article, nor is swamped by unnecessary information that still needs disambiguation; only data and information relevant to the topic of the article is made available.

### Implementation

The system comprises three parts: the Utopia Documents reader; the GPCRDB; and a module that performs the communication between the two. Users can annotate a document with GPCR-specific information with a single click, but, behind the scenes, the PDF annotation is a multi-step process:

1. Extracting the text from the PDF document

2. Identifying concepts (species, proteins, residues and mutations)

3. Creating relevant annotations based on GPCRDB data

4. Adding the annotations to the document

**Figure 1:**
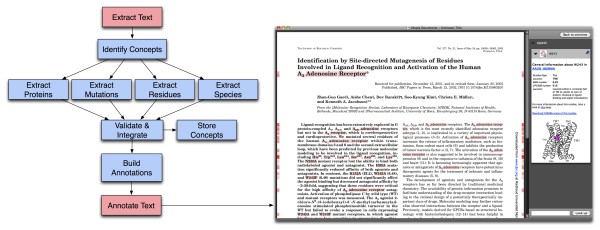
**Schematic flow-chart of the annotation process**. On the left, processes coloured red are performed by Utopia Documents, processes in blue by GPCRDB. On the right, is a screenshot of an annotated paper [29] in the Utopia Documents PDF reader, in which all annotated concepts have been highlighted by pressing the space-bar (annotations appear as red highlights, both in the body of the text and in the document margins).

1 and 4 are carried out by Utopia Documents, while 2 and 3 are performed by GPCRDB (Figure 1). The communication between the device running Utopia Documents and GPCRDB is done from within Utopia Documents by a GPCRDB-specific plugin. This plugin, written in the Python programming language, makes use of the publicly available Web service (SOAP) and Utopia's plugin-API to facilitate the communication and data exchanges between the Utopia Documents reader at the scientist's computer and the remote GPCRDB computing facilities. Within the plugin, the text is extracted from the PDF document and sent via the Web service to GPCRDB. There, the text is analysed automatically and annotations are created that are passed back to the main Utopia client to be displayed on the article as highlighted regions. More information on plugins can be obtained from the paper by Attwood *et al. *[13].

### Identifying concepts

Concepts for which we can provide information (proteins, residues and mutations) must be unambiguously identified to be able to present users with relevant information. Clearly, it is important to ensure that terms in the PDF are correctly mapped to terms in GPCRDB. Concepts must be found in the text and coupled to a unique database identifier, a process referred to as *normalization *[14,15]. In the case of a protein, this requires knowledge about the species from which it originates. The term 'rhodopsin', for example, cannot be normalised, whereas the words 'human rhodopsin' would map to the UniProtKB:Swiss-Prot identifier 'OPSD_HUMAN'. For residues and mutations, each occurrence needs to be identified and associated with one of the proteins mentioned in the text, a process called *grounding *[16-18]: e.g., 'Trp161' is meaningless, whereas 'Trp161 in human rhodopsin' maps to a unique residue in one specific protein, and is thus grounded on a normalised protein.

It is beyond the scope of this article to provide an exhaustive explanation of all algorithms and heuristics involved in normalisation and grounding. However, at the heart of both tasks lies the use of a metric based on word distances, in which combinations of terms that are close together in the text are favoured over more distant combinations. The distance metrics are combined with a number of heuristics, filters and validations. Some of the heuristics have been described elsewhere [16,18,19], while a series of related heuristics have been newly designed and implemented. In the following sections, we describe the most important steps involved in extracting and identifying proteins, residues and mutations.

### Protein identification and normalisation

Protein identification is the non-trivial task of pinpointing words that represent a gene or protein. We use a dictionary-based approach. Dictionaries are populated with gene identifiers, protein identifiers and protein descriptions from all GPCRs in GPCRDB that are present in UniProtKB:Swiss-Prot [20]. UniProtKB's Swiss-Prot component is used because of its consistent mapping between protein sequences and their descriptions. Commonly observed synonyms not present in the database-extracted list were manually added to the dictionaries. The text is scanned for gene or protein occurrences using approximate dictionary matching, allowing for some variability. Separate dictionaries are created for gene identifiers, protein identifiers and protein descriptions, each with their own scoring metric. This makes it possible to set high penalties for mismatches between reference dictionary entries and detected gene or protein identifiers, while allowing more variability (i.e., the use of both Latin and Greek characters in protein descriptions) when scoring protein descriptions, taking into account their free-text and relatively changeable nature. The Linnaeus [21] system is used to find species occurrences in the text, and to normalise these occurrences to NCBI taxonomy [22] identifiers. The normalised species occurrences are then used to complete the normalisation of the identified protein occurrences.

### Mutation and residue identification and grounding

Identifying point mutations and residues involves two subtasks. First, it is necessary to identify the mutation or residue terms discussed in an article. Second, the identified residues and mutations must be grounded.

Three entities in the text need to be related to identify point mutations: the wild type residue, the mutant residue, and the position in the sequence where the substitution occurred. Point mutations are represented in the literature in a variety of ways. The most common representation is in the form 'XnY', where × is the single-letter code for the wild-type residue, n its location and Y the single-letter code for the substituted residue (e.g., 'D98F' denotes a change from aspartic acid to phenylalanine at position 98). However, there are many variations on this theme, ranging from the use of three-letter amino acid codes, as in 'Asp98Phe' and 'Asp98 → Phe', to more exotic notations, like 'Asp98-Phe98', 'D98 to phenylalanine' and 'Asp 98 was mutated to phenylalanine'. Regular expressions are used to identify residues and mutations.

Articles about GPCRs refer to mutations and residues using one of four numbering schemes. The most frequently used are the standard sequential numbering scheme and the scheme of Ballesteros-Weinstein [23]. Other articles use the Oliveira scheme [24] (also known as the GPCRDB numbering scheme), or their own, article-specific scheme. The Ballesteros-Weinstein and Oliveira schemes are so-called general residue numbering schemes. These allow consistent residue numbering across multiple proteins, independent of their sequential numbers. The underlying principle is that residues with the same general residue number have equivalent locations in their tertiary structures and consequently in the multiple sequence alignments. In the widely used Ballesteros-Weinstein scheme, residue numbers are in the format 'TM.n', where the number before the period indicates the number of the transmembrane helix, and the number after the period indicates the residue position with respect to the most conserved residue position in the helix (e.g., '3.50' denotes residue 50 in TM domain 3).

Some articles discuss residues and mutations using an article-specific numbering scheme. Often, standard sequential residue numbers are used that do not exactly map to the protein sequence in the database. This mismatch can arise owing to inclusion of signal peptides in the sequence, different isoforms, etc. In these cases, there is an offset by which the residue numbers must be corrected to obtain a valid mapping to the reference sequences in the database. A regular expression-based approach is taken to calculate the right offsets. When multiple offsets are possible (i.e., there are multiple ways of mapping the identified residues on the sequence), the offset is chosen that is closest to the residue numbers mentioned in the text.

Residues and mutations are grounded mainly based on proximity to proteins mentioned in the text, favouring combinations that occur within the same sentence. Validation of these combinations is performed by comparing the identified residues and mutations to the sequences of the candidate protein mentions, using similar approaches to those discussed by Horn *et al. *[19]. Where general residue numbers have been used, the sequential numbers are retrieved from GPCRDB for all candidate protein mentions.

## Results

GPCRDB contains a substantial amount of information, but for this to be useful, users require more than just large collections of 'data'. For the three concepts for which we provide annotations (proteins, residues and mutations), we have selected data, links and knowledge that we believe will benefit users when reading an article, allowing them to link effortlessly from the text to all relevant information in GPCRDB. Once a paper is annotated, red marks on the side of the pages indicate where annotated concepts are located, as shown in Figure 1. The intensity of the red marks increases with the number of annotations at those positions. All annotated concepts can also be highlighted simultaneously by pressing the space-bar.

### Proteins

**Figure 2:**
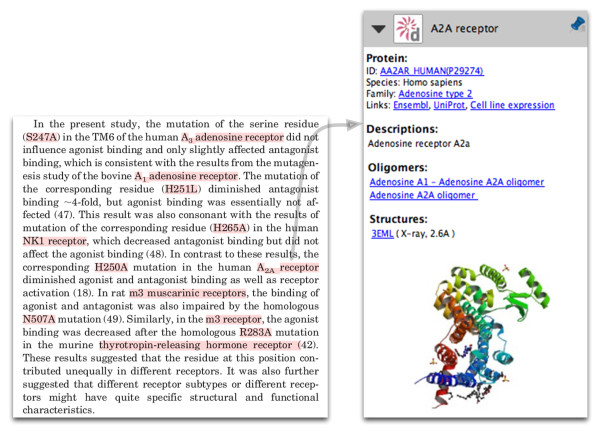
**Protein annotation of the human A2a adenosine receptor**. On the left, part of a paper by Gao et al. [29] is shown; annotated concepts are highlighted in red by pressing the space-bar. On the right, annotation for the A2a receptor (obtained by clicking on the words indicated by the grey arrow) is shown. The protein identifier and family are linked to the protein detail and protein family pages in GPCRDB, respectively. In addition, links to a number of external resources are provided. An image of the protein structure (PDB code 3EML) is shown. Although not annotated in the text, the species mentioned (human, murine, bovine, rat) are used internally for protein normalisation.

Protein annotations contain links to a number of sources (Figure 2). A link pointing to the protein detail page in GPCRDB is always present. Here, users can find information relating to the protein sequence, mutations, homology models, ligand-binding data, and so on. In addition, a link to the protein family page is provided. From here, all other protein family members can be retrieved, multiple sequence alignments can be accessed, etc.

Protein annotations also contain links to UniProtKB for a detailed report on the protein, and, if available, a link to Ensembl for the genomic view. We provide a list of synonyms for the protein names. Structural information on GPCRs is still limited, but we provide links to structures when they are available. If oligomerisation information is available, we provide links to pages that contain the relevant details.

### Residues

**Figure 3:**
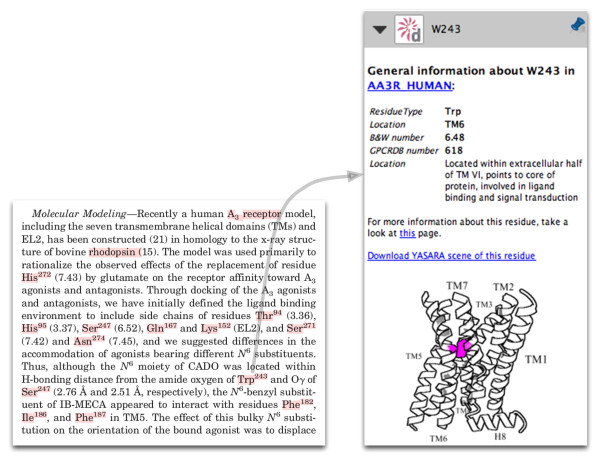
**Annotation of residue Trp243 in the human adenosine A3 receptor**. On the left, part of a paper by Gao et al. [29] is shown; annotated concepts are highlighted in red by pressing the space-bar. On the right are the residue annotations. The protein name is linked to the protein detail page in GPCRDB. Links are also provided to a page in GPCRDB listing all available information for this residue, and to a YASARA [30] scene, in which the residue is highlighted. Residue numbers are provided both in the sequential numbering scheme and in the Ballesteros-Weinstein and Oliveira (GPCRDB) schemes. A short description of the location and interactions of this residue is provided, as well as a cartoon indicating its location in the receptor structure.

Residue annotations contain various links and a selection of data pertinent to that specific residue (Figure 3).

The protein name is linked to its protein detail page in GPCRDB. The residue type is displayed, together with its residue number in several numbering schemes. General residue numbers are provided in two different schemes: the commonly used Ballesteros-Weinstein scheme, and that of Oliveira *et al. *The sequential residue number is also shown. Sequential residue numbers in GPCRDB annotations may not be identical to residue numbers described in an article. If the software has detected a mismatch and made the appropriate corrections, this will be noted in the annotation. A one-line description of the location and interactions of the residue is given, based on manual analysis of currently available protein structures. A three-dimensional cartoon image of a GPCR structure is provided, in which the position of the residue is highlighted. This allows readers to directly place the information in the article in a structural context. If the residue is located in a structurally conserved region of the Class A GPCRs, and thus has a general residue number in GPCRDB, a link is given that will create a YASARA scene containing the homology model of the protein with the residue highlighted in the structure. If the residue is part of a reported oligomerisation interface, links to more detailed descriptions of that interface are included. For each residue, a link is provided to a GPCRDB page summarising all known data and information for that residue, including information about available mutations at that position, and mutations at equivalent positions in other proteins.

### Mutations

The mutation annotations also link to the protein detail page. If GPCRDB contains information about the same mutation from other literature, links to the relevant PubMed pages are provided. This way, users can immediately look up literature that discusses the same mutation. If GPCRDB contains mutations at the same position in the same protein, but with a different mutated residue type, links to relevant GPCRDB pages are provided.

**Figure 4:**
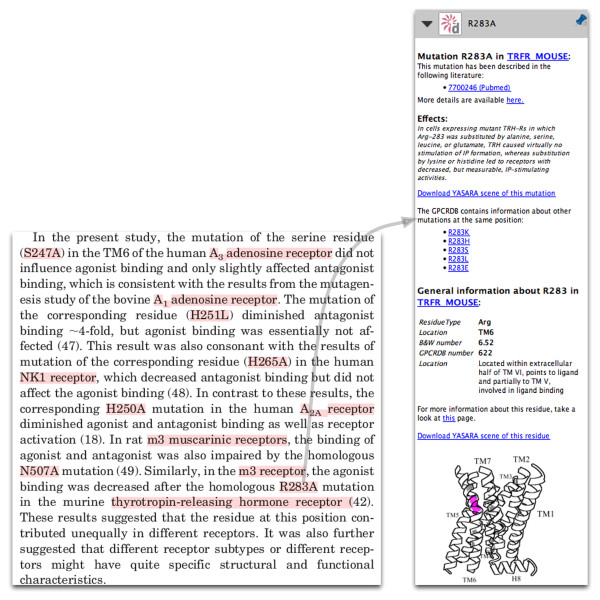
**Annotation of mutation R283A in the mouse thyrotropin-releasing hormone receptor**. On the left, part of a paper by Gao et al. [29] is shown; annotated concepts are highlighted in red by pressing the space-bar. On the right are the mutation annotations. Papers discussing the same mutation are listed, with links to PubMed. A short literature-based summary of the effects of this mutation is provided, together with links to GPCRDB pages giving details of other mutations at the same position, and to a YASARA [30] scene, in which the mutated residue is highlighted and displayed with the mutant residue type. Information about the wild-type residue is also provided, which is identical to the information displayed for residue annotations.

A link to a page that displays mutations at the same (equivalent) position in other proteins is always present. In addition to links to other literature or other mutations, GPCRDB contains a large amount of descriptive information about the effects of mutations - such information has been culled, over the years, from painstaking analysis of the literature, and manually added to the knowledgebase in the form of short summaries. Where such information is present in the GPCRDB for the mutation at hand, this information is provided automatically in the annotations displayed in the side-bar. If the mutation is located at a position in a structurally conserved region of the Class A GPCRs, and thus has a general residue number in GPCRDB, a link is given that will create a YASARA scene containing the homology model of the protein, with the wild-type and mutated residues highlighted in the structure. In addition to mutation-specific information, mutation annotations also contain general information about the mutated residue. This residue-specific annotation is identical to the information displayed within residue annotations (Figure 4).

### Community annotations

With this work, we take the integration of literature data in GPCRDB one step further. Using the PDF reader, when a mutation is identified in a scientific article that is not yet known to the GPCRDB, this mutation is automatically integrated into the GPCRDB, making this information available from both the GPCRDB website as well as from the annotations within Utopia Documents. Thus, when a user reads another article discussing a mutation at the same position, links to the newly integrated article will appear as an integral part of the annotation display, allowing immediate navigation to the appropriate literature.

In addition, behind the scenes, for all articles annotated using the reader, sentences are extracted in which mutations are mentioned. This information, which effectively provides an at-a-glance summary of the article with respect to that mutation, is stored in the GPCRDB and made available from the GPCRDB website. Because we strive to include only the most highly relevant data and information in the annotations offered by Utopia Documents, this automatically extracted text is not displayed with the PDF reader, and is only accessible through the GPCRDB website.

### Annotations and the rescue of data

**Figure 5:**
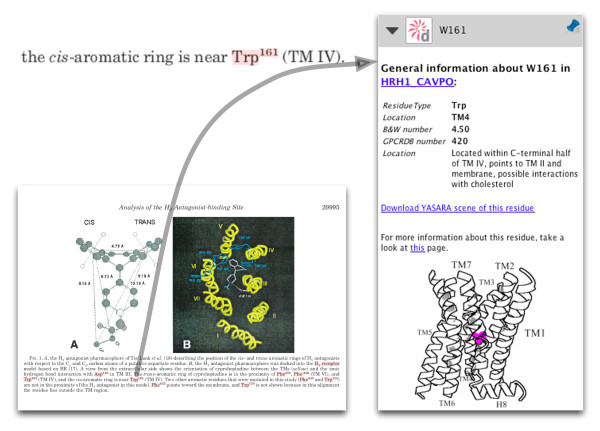
**Excerpt from the article by Wieland et al.**[27]. Trp161 is discussed as having interactions with the cis-aromatic ring of the ligand. The annotation of residue Trp161 is shown on the right as it appears in Utopia.

The integrated view of literature and data offered here is invaluable when reading articles published before the first X-ray structure of a GPCR, that of bovine rhodopsin [25], became available. Prior to that moment, researchers often interpreted their experimental results in the light of homology models. The quality of those models was usually very poor [26], and hence the interpretation of the experimental data was often far from optimal, and sometimes simply wrong. The experimental data are still valuable though; today, with more information available, scientists can re-interpret the data in light of current knowledge. As an example, Figure 5 illustrates an image from an old mutation study [27] in which the authors describe various mutations in the guinea pig histamine H1 receptor, building and validating a homology model using these data, and arguing, for example, that residue Trp161 plays an important role in receptor-ligand binding. This assumption was based on the effect of the mutation on receptor function, leading to a model in which Trp161 was (wrongly) modelled as being positioned in the ligand-binding site.

**Figure 6:**
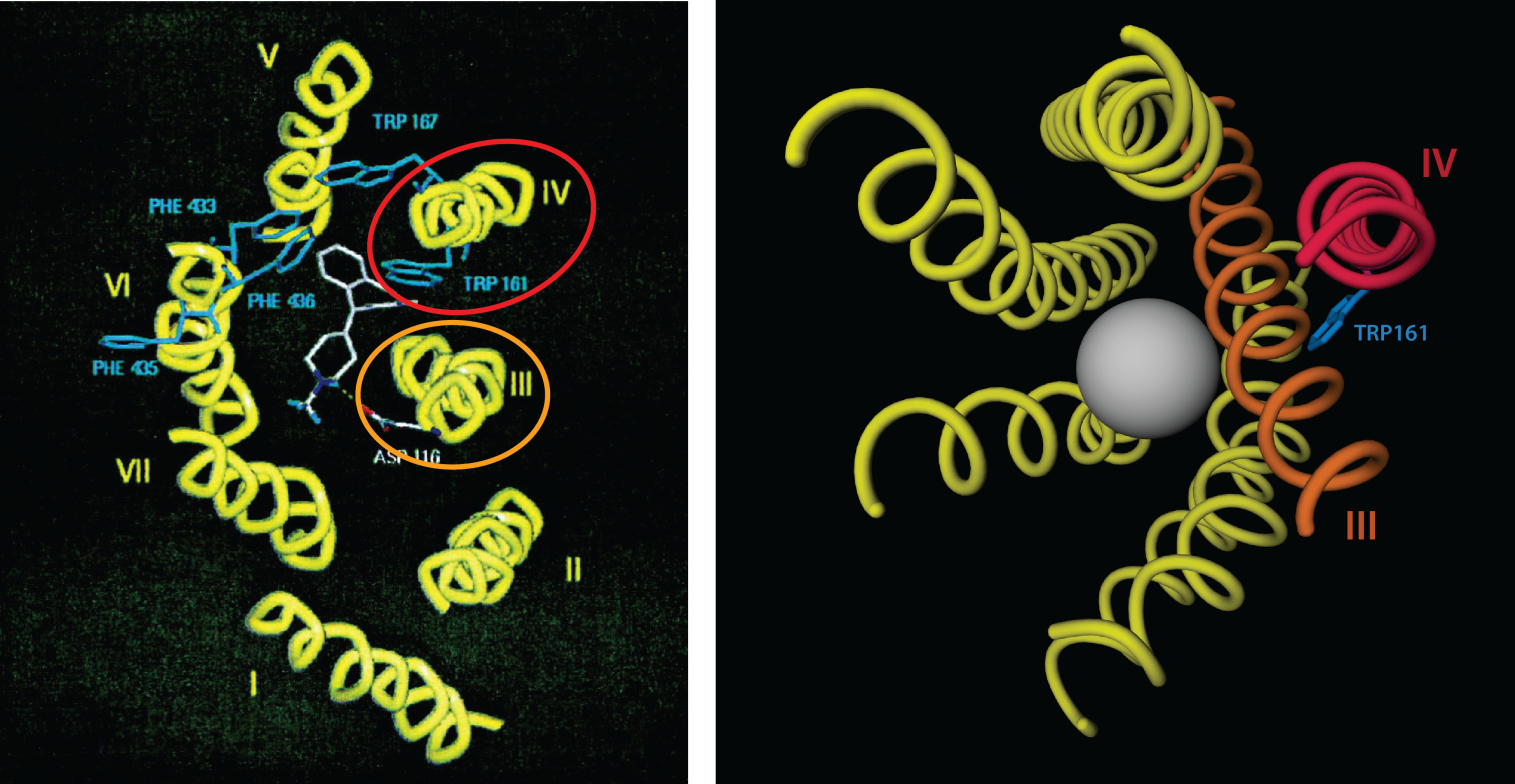
**The location of Trp161**. On the left, the model from the paper by Wieland et al. [27] to which orange and red ellipses have been added to highlight helices III and IV respectively. On the right, the model retrieved from GPCRDB, with helices III and IV drawn in orange and red. The grey sphere shows the approximate position of the ligand-binding pocket.

By contrast, the annotation indicates that this residue, located in TM IV, points towards the membrane and possibly interacts with cholesterol (Figure 5). This is a completely different view from that proposed by the authors. Looking at the model provided by GPCRDB, based on sub-family specific profiles and the latest crystal structures, it can be seen that the position of Trp161 with respect to the ligand-binding pocket is completely obscured by TM III and hence that a direct role in receptor-ligand binding is highly unlikely (Figure 6).

The point here is not that the Wieland model is incorrect; as stated before, most models were wrong before the structure of bovine rhodopsin was published. What is important is that this software can be used to detect these types of historical artifacts, relieving scientists of the task of validating the results of 'old' literature against current knowledge. It is important to emphasise that the published experimental data are still valuable, but need new interpretation: in a sense, the software rescues old data from articles that contain interpretations that we now know to be incorrect. With this tool, therefore, scientists can now focus on providing new views on experimental data in the light of current knowledge. For example, knowing that Trp161 is conserved and interacts with cholesterol in some receptor structures allows for the hypotheses that this residue is functionally important for dimer formation, potentially through cholesterol. Other hypotheses can be imagined too, but the only thing that seems certain is that Trp161 is not directly involved in ligand binding.

### Applications

The system presented here is likely to be useful in a number of different scenarios. For example, for biocurators, it offers a rapid means of accessing reliable, contextualised GPCR-relevant data: this would not only benefit future curators of GPCR-specific resources like GPCRDB, but would also facilitate the work of those who annotate more general repositories, such as UniProtKB:Swiss-Prot or InterPro [28]. For journal editors, it offers a swift route to 'gold standard' GPCR data that they may officially endorse as part of their manuscript mark-up process. For life scientists and bioinformaticians in general, and for pharmacologists and target-discovery researchers in particular, it offers a convenient way to keep up with GPCR literature, and to find new articles and new links that they might otherwise have overlooked. For users of GPCRDB, it offers a swifter, more targeted and hence more convenient navigation system compared to simple Web-based browsing of the resource. Many other use cases are likely as the system is generalised to other superfamilies.

## Conclusions

The work presented here was developed in response to the need to achieve tighter coupling between published articles and their underlying data as a crucial step towards knowledge discovery. The strength of our approach lies in its ability to present users (life science researchers, bioinformaticians, researchers in the pharmaceutical industry, journal editors, and so on) with current, integrated, validated, internally consistent data and information in the context of literature being read. The annotations provided are extensive and detailed, and, importantly, include concepts such as 'residue A interacts with the ligand', 'residue B is implicated as playing a role in oligomeric interactions', 'residue C is located in TM4 and points to the lipid environment', 'residue D is conserved in its sub-family', etc. A substantial amount of knowledge is sequestered in such annotations. Taking mutation annotations as an example, although trivial at first glance, the mapping of sequential residue numbers to those of general residue numbering schemes saves researchers large amounts of time in looking up the residue mentioned in the database and finding its associated general residue number. In order to reliably transfer information between different proteins, this is a necessary but very time-consuming process. The structural information consists of data from a variety of different manually- or automatically-generated sources, and provides scientists with a structural perspective on the concepts in the text, making it much easier to critically assess the authors' interpretations of experimental results, which is crucially important when reading old literature. Linking mutation data to other articles in which similar mutations have been discussed saves users considerable effort. This is an onerous task to perform by hand, owing to the necessary conversions between various numbering schemes and residue numbering offsets. To summarise, this work helps GPCR researchers to optimally extract new knowledge from scientific literature by automatically putting data from a PDF article in the wider context of the total body of knowledge related to GPCRs.

### Availability and requirements

Project name: GPCR-specific PDF reader

Project home page: http://www.gpcr.org/7tm

Operating systems: Mac OS (10.5 or newer), Windows (XP or newer), Ubuntu linux (10.4 or newer), Debian linux (6.0 or newer)

Programming language: C++, Java, Python

Other requirements: None

License: http://www.getutopia.com/documents/License.txt

Any restrictions to use by non-academics: None

## Abbreviations

MCSIS: Molecular Class-Specific Information System; GPCR: G Protein-Coupled Receptor; PDF: Portable Document Format; SOAP: Simple Object Access Protocol

## Authors' contributions

BV drafted the manuscript and wrote the GPCRDB-specific software. BV, DT, PM and SP participated in the writing of the Utopia Documents plugin. DT, PM and SP wrote the Utopia Documents software and adapted it to be used with the GPCRDB plugin. GV supervised the project. GV & TA helped write the manuscript and draft Figure 6. All authors have read and approved the final manuscript.
